# Identification of Neurotensin Receptor Expressing Cells in the Ventral Tegmental Area across the Lifespan

**DOI:** 10.1523/ENEURO.0191-17.2018

**Published:** 2018-02-12

**Authors:** Hillary L. Woodworth, Patricia A. Perez-Bonilla, Bethany G. Beekly, Trevor J. Lewis, Gina M. Leinninger

**Affiliations:** 1 Department of Physiology, Michigan State University, East Lansing, MI 48824; 2 Neuroscience Program and Department of Pharmacology and Toxicology, Michigan State University, East Lansing, MI 48824

**Keywords:** brain, developmental, dopamine, dual recombinase, glia, mesolimbic

## Abstract

Neurotensin (Nts) promotes activation of dopamine (DA) neurons in the ventral tegmental area (VTA) via incompletely understood mechanisms. Nts can signal via the G protein-coupled Nts receptors 1 and 2 (NtsR1 and NtsR2), but the lack of methods to detect NtsR1- and NtsR2-expressing cells has limited mechanistic understanding of Nts action. To overcome this challenge, we generated dual recombinase mice that express FlpO-dependent Cre recombinase in NtsR1 or NtsR2 cells. This strategy permitted temporal control over recombination, such that we could identify NtsR1- or NtsR2-expressing cells and determine whether their distributions differed between the developing and adult brain. Using this system, we found that NtsR1 is transiently expressed in nearly all DA neurons and in many non-DA neurons in the VTA during development. However, NtsR1 expression is more restricted within the adult brain, where only two thirds of VTA DA neurons expressed NtsR1. By contrast, NtsR2 expression remains constant throughout lifespan, but it is predominantly expressed within glia. Anterograde tract tracing revealed that NtsR1 is expressed by mesolimbic, not mesocortical DA neurons, suggesting that VTA NtsR1 neurons may represent a functionally unique subset of VTA DA neurons. Collectively, this work reveals a cellular mechanism by which Nts can directly engage NtsR1-expressing DA neurons to modify DA signaling. Going forward, the dual recombinase strategy developed here will be useful to selectively modulate NtsR1- and NtsR2-expressing cells and to parse their contributions to Nts-mediated behaviors.

## Significance Statement

The activation of ventral tegmental area (VTA) dopamine (DA) neurons is disrupted in many neuropsychiatric diseases. The neuropeptide neurotensin (Nts) activates DA neurons by incompletely understood receptor mechanisms, which may be useful to modify aberrant VTA DA signaling in disease states. Using new mouse models, this study demonstrates that Nts receptor subtypes are differentially expressed in the VTA. In particular, one Nts receptor subtype is expressed predominantly on VTA DA neurons, while glial cells express a different receptor subtype. Thus, this work reveals the cellular mechanism by which Nts directly acts on DA neurons and may guide development of Nts-mediated therapies to improve DA signaling in neuropsychiatric diseases.

## Introduction

The neuropeptide, neurotensin (Nts), is expressed throughout the central nervous system and has been linked to numerous physiologic and behavioral processes including feeding, body temperature, nociception, emotional state, and sleep (Bissette et al., 1982; Remaury et al., 2002; Fitzpatrick et al., 2012; Kleczkowska and Lipkowski, 2013; Boules et al., 2014; Woodworth et al., 2017b). While the precise neural circuits by which Nts acts to modify behavior remain undetermined, it is well-established that Nts modulates dopamine (DA) transmission. Nts signaling may therefore be a target of interest for the treatment of diseases linked with altered DA signaling, including Parkinson’s disease, schizophrenia, drug addiction, and obesity (Cáceda et al., 2006; Boules et al., 2014).

Nts signals via the G protein-coupled Nts receptors 1 and 2 (NtsR1 and NtsR2), which are both expressed in the brain and have been implicated in Nts-mediated behaviors (Tanaka et al., 1990; Vita et al., 1993; Chalon et al., 1996; Mazella et al., 1996). Nts also binds the intracellular receptor, NtsR3/sortilin, which may regulate recycling and sorting of Nts (Mazella et al., 1998; Kleczkowska and Lipkowski, 2013). Studies using pharmacological antagonists and mice null for NtsR1 or NtsR2 suggest that each receptor isoform mediates distinct aspects of physiology, and hence that they may be expressed on different cell types (Schroeder and Leinninger, 2017). The lack of methods to detect and manipulate NtsR1- or NtsR2-expressing cells, however, has limited understanding of the cellular mechanisms by which Nts regulates behavior. *In situ* hybridization (ISH) and autoradiography methods to detect *Ntsr1* indicate that it is expressed robustly within the ventral tegmental area (VTA) of adult animals (Nicot et al., 1994; Alexander and Leeman, 1998; Lein et al., 2007). Similar techniques reveal diffuse expression of *Ntsr2* throughout the brain that may be within both neurons and glia (Nouel et al., 1997; Sarret et al., 1998; Walker et al., 1998; Sarret et al., 2003). Interestingly, the expression patterns of NtsR1 and NtsR2 in the brain may also vary with age. For example, *Ntsr1* is transiently upregulated during gestation and peaks shortly after birth, but is subsequently downregulated as animals reach maturity, with high levels persisting in the VTA (Palacios et al., 1988). By contrast, *Ntsr2* expression is initially low and gradually increases with age (Sarret et al., 1998; Lépée-Lorgeoux et al., 1999). Taken together, these data indicate that *Ntsr1* and *Ntsr2* have distinct expression patterns that vary across the lifespan, and may be found on different cell types within the nervous system.

The differences in *Ntsr1* and *Ntsr2* expression suggest that each isoform may regulate distinct aspects of developmental and adult physiology. Indeed, previous work demonstrates that central Nts promotes DA release, locomotor activity, hypothermia, anorexia, and reward via NtsR1 (Pettibone et al., 2002; Remaury et al., 2002; Leonetti et al., 2004; Kim et al., 2008; Kempadoo et al., 2013; Opland et al., 2013; Rouibi et al., 2015), whereas NtsR2 may confer the pain-reducing effects of Nts (Remaury et al., 2002; Maeno et al., 2004; Roussy et al., 2010; Kleczkowska and Lipkowski, 2013). However, the evidence for distinct roles of NtsR1 and NtsR2 is not entirely consistent and has been complicated by methodological limitations. For example, the commonly used NtsR1-selective antagonist SR48692 also acts as an agonist at NtsR2 (Botto et al., 1997; Vita et al., 1998; Yamada et al., 1998), while a potential compound to selectively antagonize NtsR2 has only recently been developed (Thomas et al., 2016). NtsR1 and NtsR2 knock-out mice have also been used to examine the specific roles of each receptor, but developmental deletion in these models may lead to compensatory changes that mask normal action of the Nts system. (Pettibone et al., 2002; Remaury et al., 2002; Kim et al., 2008; Liang et al., 2010). Thus, while NtsR-selective pharmacologic agents and knock-out models have added to understanding of central Nts action, developing methods to visualize and manipulate select NtsR1 or NtsR2 populations *in vivo* is essential to deciphering the neural circuits and physiology regulated by each receptor.

To address this challenge, we developed dual recombinase knock-in mouse models in which FlpO is required to induce IRES-Cre in cells that express NtsR1 or NtsR2. Cre-mediated recombination can be used to induce reporter or effector proteins in these cells to permit their detection, and indeed Cre-driver lines have proven to be reliable reagents to identify genetically specified cell populations (Krashes et al., 2011; Leinninger et al., 2011; Vong et al., 2011). As NtsR1 and NtsR2 expression varies with age (Palacios et al., 1988; Lépée-Lorgeoux et al., 1999), we engineered FlpO-dependent Cre expression in NtsR1 and NtsR2 cells, allowing for temporal control over recombination by inducing FlpO expression at defined time points (either embryogenesis or adulthood). Given the well-established description of Nts as a modulator of DA signaling, but the lack of understanding of which VTA cells mediate it, we used these mice to define the cellular distribution of NtsR1 and NtsR2 within the VTA.

## Materials and Methods

### Generation of *NtsR1^NEO-Cre^* and *NtsR2^NEO-Cre^* knock -in mice

We have tested commercial antibodies against NtsR1 or NtsR2, but they did not yield labeling consistent with the ISH distribution of NtsR1 and NtsR2 in mouse brain (Lein et al., 2007), or sufficient to discern which cells express these receptor isoforms. (Antibodies tried for NtsR1 include Santa Cruz sc-374492 and aNeuromics GP14020. Antibodies tried for NtsR2 were Santa Cruz sc-67011 and Neuromics RA17100). We therefore generated knock-in mice to permit facile identification of NtsR1- and NtsR2-expressing cells. *NtsR1^NEO-Cre^* and *NtsR2^NEO-Cre^* targeting vectors were generated by inserting an *IRES-Cre* between the stop codon and the polyadenylation site of the sequence encoding the 3’ end of the mouse *NtsR1* gene, with an *frt-*flanked *NEO* cassette placed upstream of the *IRES-Cre*. The linearized targeting vector was electroporated into mouse R1 embryonic stem (ES) cells (129sv background) and cells were selected with G418. DNA from ES cell clones was analyzed via qPCR for loss of homozygosity using Taqman primer and probes for the genomic *NtsR1* or *NtsR2* insertion sites (*NtsR1*: forward, TCTGATGTTGGACTTGGGTTC; reverse, TCTGATGTTGGACTTGGGTTC; probe, TCTGATGTTGGACTTGGGTTC and *NtsR2*: forward, ACCCATCAGATAAGCCATGC; reverse, GTGGGAAGTTGAGGGCAG; probe, GTCTAAGCGGACCTACTGACCCA). *NGF* was used as a copy number control (Soliman et al., 2007). Putative positive ES clones were expanded, confirmed for homologous recombination by Southern blot and injected into mouse C57BL/6 blastocysts to generate chimeras. Chimeric males were mated with C57BL/6 females (The Jackson Laboratory), and germline transmission was determined initially via progeny coat color, then confirmed via conventional PCR for *IRES-Cre*.

### Breeding and genotyping

Mice were bred and housed in a 12/12 h light/dark cycle with *ad libitum* access to water and food (Harlan Teklad #7913). All procedures were approved by the Institutional Animal Care and Use Committee at Michigan State University in accordance with Association for Assessment and Accreditation of Laboratory Animal Care and National Institutes of Health guidelines. Heterozygous *NtsR1^NEO-Cre^* and *NtsR2^NEO-Cre^*mice were bred either to FlpO deleter mice (Jax Stock #012930), a Cre-inducible Rosa^eGFP-L10a^ reporter (Krashes et al., 2014) that was graciously provided to us by David P. Olson (University of Michigan), or to C57/Bl6 wild-type mice to maintain the lines.

#### Developmental model

*NtsR1^NEO-Cre^* and *NtsR2^NEO-Cre^* mice were bred to a FlpO deleter line and progeny that inherited the FlpO allele (*NtsR1^ΔNEO-Cre^* and *NtsR2^ΔNEO-Cre^*) were subsequently mated with a Cre-inducible *Rosa^eGFP-L10a^* reporter, generating *NtsR1^Dev^;GFP* and *NtsR2^Dev^;GFP* mice. Progeny that were heterozygous for both *IRES-Cre* and *GFP* alleles were used for analysis.

#### Adult model

*NtsR1^NEO-Cre^* and *NtsR2^NEO-Cre^*mice were mated directly to Cre-inducible *Rosa^eGFP-L10a^* animals, producing heterozygous *NtsR1^NEO-Cre^;GFP* and *NtsR2^NEO-Cre^;GFP* progeny. These animals were then injected with FlpO adenovirus in adulthood (see below) to generate *NtsR1^Adult^;GFP* and *NtsR2^Adult^;GFP* study mice. All animals were genotyped by standard PCR using the following primer sequences. *IRES-Cre*: forward, GGACGTGGTTTTCCTTTGAA and reverse, AGGCAAATTTTGGTGTACGG. *Rosa26^EGFP-L10a^*: mutant forward, TCTACAAATGTGGTAGATCCAGGC; wild type forward, GAGGGGAGTGTTGCAATACC; common, CAGATGACTACCTATCCTCCC. *FlpO*: mutant, GCGAAGAGTTTGTCCTCAACC; common, GCG AAG AGT TTG TCC TCA ACC; wild type, GGAGCGGGAGAAATGGATATG. Adult male and female mice of each model were studied.

### Surgery

Adult *NtsR1^Adult^;GFP* and *NtsR2^Adult^;GFP* mice received a presurgical injection of carprofen (5 mg/kg, s.c.) and were anesthetized with 3–4% isoflurane/O_2_ in an induction chamber before being placed in a stereotaxic frame (Kopf). Under 1–2% isoflurane, access holes were drilled in the skull allowing a guide cannula with stylet (Plastics One) to be lowered into the lateral ventricles (A/P: –0.22, M/L: ±1.0, D/V: –2.0). Mice were bilaterally injected with 1 µl FlpO adenovirus (Vector Biolabs), which was infused at a rate of 1 µl/min. The animals recovered for 10 d before perfusion to permit sufficient time for FlpO-mediated excision of the *frt*-flanked *NEO* cassette and GFP expression. For tracing studies, *NtsR1^ΔNEO-Cre^;GFP* mice were injected unilaterally in the VTA (A/P: –3.2, M/L: ±0.48, D/V: –4.65) with 75–100 nl of Ad-syn-mCherry, an adenovirus expressing a Cre-dependent synaptophysin-mCherry fusion protein (Opland et al., 2013). Mice recovered for either 7–10 d or four weeks after surgery to allow for Cre-mediated recombination and synaptophysin-mCherry expression at presynaptic terminals. The Ad-Syn-mCherry tracing system has been validated previously, including verification of its Cre dependence to induce synaptophysin-mCherry expression (Woodworth et al., 2017a).

### Perfusion and immunofluorescence

Mice were treated with a lethal dose of intraperitoneal pentobarbital followed by transcardial perfusion with 10% neutral-buffered formalin (Fisher Scientific). Brains were removed, postfixed in 10% formalin overnight at 4°C and dehydrated with 30% sucrose in PBS for 2–3 d. Then, each brain was sectioned into four series of 30-µm coronal sections, each of which provides a representative survey of the entire brain. For experiments, a single series of brain sections from each mouse was analyzed by immunofluorescence or immunohistochemistry as previously described (Leinninger et al., 2011; Opland et al., 2013). For characterization of NtsR1-GFP and NtsR2-GFP cells, sections were exposed to chicken anti-GFP (1:2000, Abcam, catalog #ab13970 RRID: AB_300798), S100 β (1:1000, Abcam, catalog #ab52642, RRID: AB_88246), NeuN (1:1000, MilliporeSigma, catalog #MAB377, RRID: AB_2298772), and mouse anti-TH (1:1000, Millipore, catalog #MAB318 RRID: AB_2201528), followed by incubation with species-specific secondary antibodies conjugated to Alexa Fluor 488 or 568 fluorophores (Life Technologies catalog #A10037 RRID: AB_2534013 and Jackson ImmunoResearch catalog #703-545-155 RRID: AB_2340375). Brains were analyzed using an Olympus BX53 fluorescence microscope outfitted with FITC and Texas Red filters. Microscope images were collected using Cell Sens software and a Qi-Click 12 Bit cooled camera, and images were analyzed using Photoshop software (Adobe). Masks were applied to images to enhance brightness and/or contrast in Photoshop, and in every case, the mask was applied uniformly to the entire image.

### Cell counts

Brains were sectioned into four series of 30-µm coronal sections, each of which provides a representative survey of the entire brain. Only one series of the four was directly analyzed in counting studies to determine the number of neurons in the VTA. We then multiplied the number of counted neurons by four to estimate the total number of neurons in the entire VTA. For each mouse, multiple 10× images were captured of each VTA-containing brain section and were assigned a bregma coordinate based on the atlas of Paxinos and Franklin (Paxinos and Franklin, 2001). We chose 10× magnification because an entire VTA hemisphere can be captured in a single 10× image, which allowed us to identify multiple landmarks within the image used to define the borders of the VTA. 10× images spanning the entire rostro-caudal axis of the VTA (roughly bregma –3.90 to –3.00 mm) were then compiled using Photoshop, so that images from each animal were arranged side-by-side according to bregma level. The Paxinos atlas and cellular architecture was used to identify the midline and interpeduncular nucleus (ip) that comprise the medial boundary of the VTA, and the sites of the medial lemniscus (ml), and substantia nigra (SN) were used to determine the lateral border of the VTA. Where possible, TH immunoreactivity was used to define the superior and inferior borders of the VTA, as well as to dissociate TH neurons of the VTA and SN (as the TH+ neurons in the SN tend to be smaller and more tightly packed compared to those of the VTA). Together, these criteria were used to draw the borders of the VTA onto each image. Once the VTA boundaries were defined, five representative VTA sections spanning bregma –3.90 to –3.00 mm were selected from each mouse, and were used to quantify GFP or GFP/TH+ neurons. Sections were ∼0.15–0.20 mm apart from each other to maintain equal coverage of the VTA between mice. Images chosen for counting were digitally magnified in Photoshop to 20× or higher, so that the experimenter could easily identify and count the number of GFP or GFP/TH+ double-positive neurons. For GFP counts: *NtsR1^Dev^ n* = 3, *NtsR2^Dev^ n* = 3, *NtsR1^Adult^ n* = 3, *NtsR2^Adult^ n* = 6. To determine the percentage of all VTA TH+ neurons that colocalize with NtsR1-GFP or NtsR2-GFP: *NtsR1^Dev^ n* = 3; *NtsR2^Dev^ n* = 3; *NtsR1^Adult^ n* = 3; *NtsR2^Adult^ n* = 6.

### Statistics

All data were analyzed in Prism 6 (GraphPad) using unpaired *t* tests. Bar graphs represent mean ± SEM. See Table 1 for power analysis.

**Table 1.** Statistical table

Figure	Data structure	Statistical test	Power
a Fig. 2*G*	Normal distribution	Unpaired *t* test	1.00
b Fig. 2*H*	Normal distribution	Unpaired *t* test	1.00
c Fig. 5*E*	Normal distribution	Unpaired *t* test	0.99
d Fig. 5*F*	Normal distribution	Unpaired *t* test	1.00
e Fig. 5*G*	Normal distribution	Unpaired *t* test	1.00
f Fig. 5*H*	Normal distribution	Unpaired *t* test	1.00

## Results

### Dual recombinase strategy to label NtsR1- and NtsR2-expressing cells

**Figure 1.**
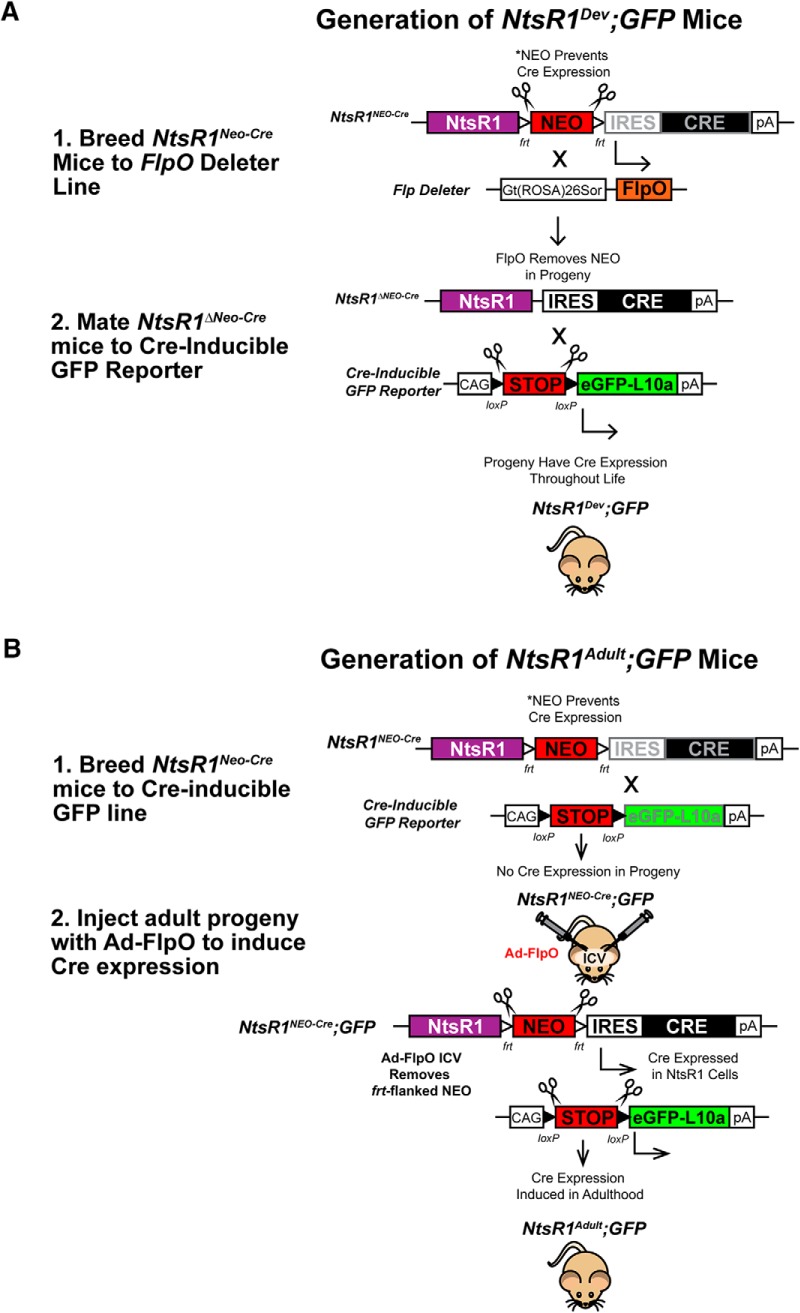
Generation of mouse models to identify developmental versus adult expression patterns of NtsR1 and NtsR2. ***A***, Schematic demonstrating how constitutive Cre expression is induced in *NtsR1^Neo-Cre^* mice, resulting in GFP labeling of any neuron that expresses NtsR1 throughout the lifespan (*NtsR1^Dev^* model). ***B***, Schematic depiction of how Cre expression is suppressed until adulthood in *NtsR1^Neo-Cre^* mice, resulting in GFP labeling restricted to cells actively expressing NtsR1 in adult animals (*NtsR1^Adult^*model). The same strategies depicted in ***A***, ***B*** were used in *NtsR2^Neo-Cre^* mice.

To identify NtsR1 or NtsR2 cells “on command,” we generated knock-in mouse models that express Cre in NtsR1 or NtsR2 cells only after FlpO-mediated recombination. To do this, we inserted an *frt*-flanked *NEO* cassette upstream of an *IRES-Cre* sequence and cloned it into the non-coding region of the *NtsR1* or *NtsR2* genomic sequences; we refer to these as *NtsR1^NEO-Cre^* and *NtsR2^NEO-Cre^* mice (Fig. 1). The *frt*-flanked *NEO* cassette blocks Cre expression unless *NEO* is removed, thus *NtsR1^NEO-Cre^* and *NtsR2^NEO-Cre^* mice lack Cre expression until exposure to FlpO.

To reveal cells that express NtsR1 and NtsR2 during development, we crossed *NtsR1^NEO-Cre^* and *NtsR2^NEO-Cre^* mice to a FlpO deleter line, producing progeny that lack the *frt*-flanked *NEO* cassette and thus produce *Cre* whenever *Ntsr1* or *Ntsr2* is transcribed throughout the lifespan (*NtsR1^ΔNEO-Cre^* and *NtsR2^ΔNEO-Cre^* mice). The *NtsR1^ΔNEO-Cre^* and *NtsR2^ΔNEO-Cre^* mice were subsequently bred to a Cre-inducible Rosa^eGFP-L10a^ reporter line; in progeny heterozygous for each allele, any cell that expresses NtsR1 or NtsR2 will undergo Cre-mediated recombination to express GFP. Notably, the recombination is permanent, so GFP labeling persists even in cells that cease to express *Ntsr1* or *Ntsr2.* This model enables us to visualize any cells that expressed *Ntsr1* or *Ntsr2* from conception onward, and we refer to these as *NtsR1^Dev^;GFP* and *NtsR2^Dev^;GFP* mice (Fig. 1*A*).

To study the adult expression pattern of *Ntsr1* and *Ntsr2*, we bred *NtsR1^NEO-Cre^* and *NtsR2^NEO-Cre^* mice to the Cre-inducible Rosa^eGFP-L10a^ reporter. In this case, the progeny (*NtsR1^NEO-Cre^*;*GFP* and *NtsR2^NEO-Cre^;GFP* mice) carry the *GFP* allele, but no reporter is expressed because the *frt*-flanked *NEO* cassette suppresses *Cre* expression. These mice were maintained in our colony until they reached adulthood, then they were injected with an adenovirus expressing FlpO recombinase (Ad-FlpO) into the lateral ventricles to permit viral spread and vector expression throughout the adult brain (Ghodsi et al., 1999; Gholizadeh et al., 2013). The subsequently expressed FlpO excises the *frt*-flanked *NEO*, which permits Cre-mediated GFP expression only in cells that actively express *Ntsr1* or *Ntsr2*, allowing us to visualize the adult expression patterns of these receptor subtypes (*NtsR1^Adult^;GFP* and *NtsR2^Adult^;GFP* mice; Fig. 1*B*).

### Distribution and morphology of NtsR1 and NtsR2 in the VTA

**Figure 2.**
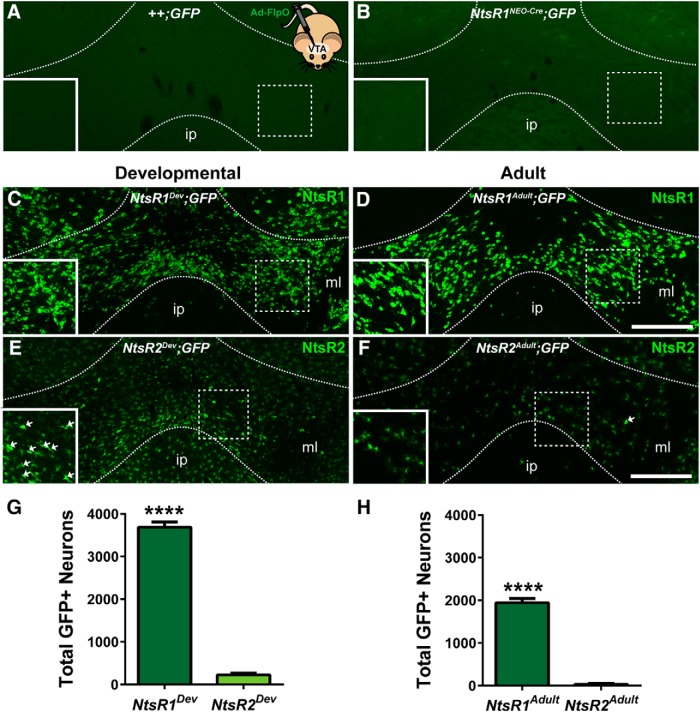
Developmental versus adult expression of NtsR1 and NtsR2 in the VTA. ***A***, No GFP is observed in the VTA of Rosa^eGFP-L10a^ reporter mice lacking Cre *(++;GFP* mice), even after Ad-FlpO injections, confirming that only Cre can drive GFP expression in this line**. *B***, *NtsR1^NEO-Cre^*;*GFP* mice do not exhibit any GFP in the VTA, suggesting that the frt-flanked *NEO* cassette effectively blocks Cre expression and thus Cre-mediated GFP expression. ***C–H***, *NtsR1^NEO-Cre^*and *NtsR2^NEO-Cre^*mice were bred to a Cre-inducible Rosa^eGFP-L10a^ reporter line and Cre expression was induced constitutively from conception (developmental expression) or only in adulthood. ***C***, ***D***, Developmental and adult distributions of NtsR1 cells. ***E***, ***F***, Developmental and adult distributions of NtsR2 cells. GFP+ cells have either neuronal or glial morphology (white arrows). Images ***A–F*** are shown at the same magnification, and scale bars represent 200 µm. Insets are digital enlargements of the areas within dashed boxes. ***G***, ***H***, Total number of GFP+ NtsR1 or NtsR2 neurons in developmental and adult models in the VTA (*NtsR1^Dev^ n* = 3; *NtsR2^Dev^ n* = 3; *NtsR1^Adult^ n* = 3; *NtsR2^Adult^ n* = 6). Each bar represents mean ± SEM, and data were analyzed by unpaired *t* tests, *p* < 0.0001 for both **G**_a_ and **H**_b_.

We first examined the mouse lines used for these studies to verify their Cre-dependent regulation of GFP expression. This is vital, because any nonspecific GFP expression could lead to over interpretation of the distribution and/or number of NtsR1- or NtsR2-expressing cells in the brain, and obscure subsequent analyses. As a first step, we assessed any potential “leakiness” of the Rosa^eGFP-L10a^ reporter line, and whether its GFP expression is specifically Cre dependent. Indeed, we found that Rosa^eGFP-L10a^ reporter mice lacking Cre (*++;GFP* mice) do not exhibit GFP expression in the VTA or anywhere in the brain, even after injected with Ad-FlpO to permit expression of the FlpO recombinase (Fig. 2*A*); thus, Cre recombinase is necessary to drive GFP expression in this line. Next, we assessed any potential leakiness in NEO-Cre containing mice crossed onto the Rosa^eGFP-L10a^ reporter line, which are the basis of the “Adult” models for identifying mature NtsR1- or NtsR2-expressing cells. Per our design (Aim 1B), we predicted that the frt-flanked NEO cassette should effectively block Cre expression and thus Cre-mediated GFP expression. Indeed we found no GFP expression in the VTA or anywhere in the brain of *NtsR1^NEO-Cre^*;*GFP* mice (Fig. 2*B*) or *NtsR2^NEO-Cre^*;*GFP* mice (data not shown). Taken together, these controls verify that GFP expression in our models is explicitly Cre dependent. Hence, any GFP observed in our studies is specifically due to Cre-mediated recombination in NtsR1 or NtsR2 cells, and can be trusted to identify them.

We next used the dual recombinase mouse models that we generated to visualize the distributions of NtsR1- and NtsR2-expressing cells in the VTA, and also whether they differed between developmental and adult stages. First, analysis of *NtsR1^Dev^;GFP* mice revealed a wide-spread, dense population of GFP+ neurons in the VTA (Fig. 2*C*) and throughout the brain (data not shown). Despite many GFP-labeled neurons, we ruled out the possibility of ectopic expression via lack of GFP in regions that are thought to have minimal NtsR1 expression at any point in life, including the ip (Geisler et al., 2006; see ip in Fig. 2*C*). By contrast, *NtsR1^Adult^;GFP* mice had far fewer GFP+ neurons that were primarily restricted to the VTA (Fig. 2*D*) and SN (data not shown). These findings are consistent with previous work showing that central *NtsR1* expression peaks during gestation, but subsequently decreases and only remains prominent in select regions, including the VTA (Palacios et al., 1988; Lépée-Lorgeoux et al., 1999).

Analysis of NtsR2-reporter mice revealed two morphologically distinct populations of GFP-labeled cells in the VTA. A few GFP+ cells displayed clear neuronal features (Fig. 2*E*, white arrows) but the vast majority of GFP-labeled cells in both developmental and adult models were detected in cells with diffuse, stellate morphology indicative of glial cells (Fig. 2*E*). This finding supports previous work suggesting that NtsR2 is expressed on astrocytes (Nouel et al., 1997, 1999; Yamauchi et al., 2007; Woodworth et al., 2017a). GFP-expressing cells with neuronal morphology were sparse in both *NtsR2^Dev^;GFP* and *NtsR2^Adult^;GFP* models but slightly more of these putative neurons were detected in *NtsR2^Dev^;GFP* mice (Fig. 2*E*,*F*). When quantified, we found that developmental NtsR1-GFP+ neurons outnumber NtsR2-GFP+ neurons ∼18–1 (Fig. 2*G*; Table 1). While the total number of NtsR1-GFP+ neurons in the adult model is about half of that observed in the developmental model, adult NtsR1-GFP+ neurons outnumber NtsR2-GFP+ neurons by 60–1 (Fig. 2*H*; Table 1). Collectively, these data indicate that NtsR1 is the predominant isoform expressed on VTA neurons in development and adulthood, while NtsR2 is primarily expressed on cells with glial morphology. Furthermore, our findings reveal that many cells express NtsR1 and NtsR2 at some stage of development, but in the adult brain NtsR1 expression is confined to a subset of VTA cells.

### Glial and neuronal markers distinguish NtsR1 and NtsR2 cells in the VTA

**Figure 3.**
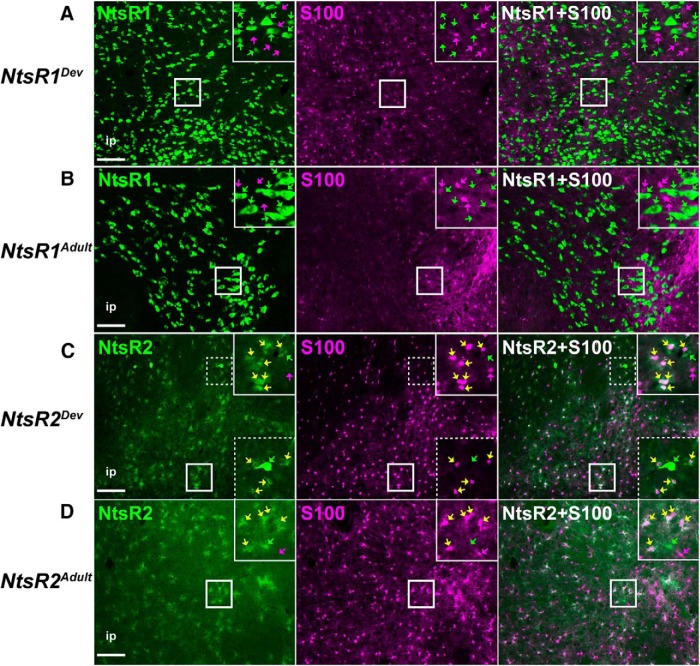
NtsR1 and NtsR2 colocalization with the glial marker S100 in development compared to adulthood. Representative images showing S100 coexpression in the VTA with (***A***) developmental NtsR1-GFP+ neurons, (***B*)** adult NtsR1-GFP+ cells, (***C***) developmental NtsR2-GFP+ cells, amd (***D***) adult NtsR2-GFP+ cells. Yellow arrows: S100/GFP+ colocalized cells, indicating NtsR-containing cells that are glia; green arrows: GFP+ only cells and magenta arrows = S100+ only cells. Scale bars: 100 µm. Insets are digital enlargements of the indicated boxed areas. *NtsR1^Dev^ n* = 3; *NtsR2^Dev^ n* = 3; *NtsR1^Adult^ n* = 3; *NtsR2^Adult^ n* = 3.

**Figure 4.**
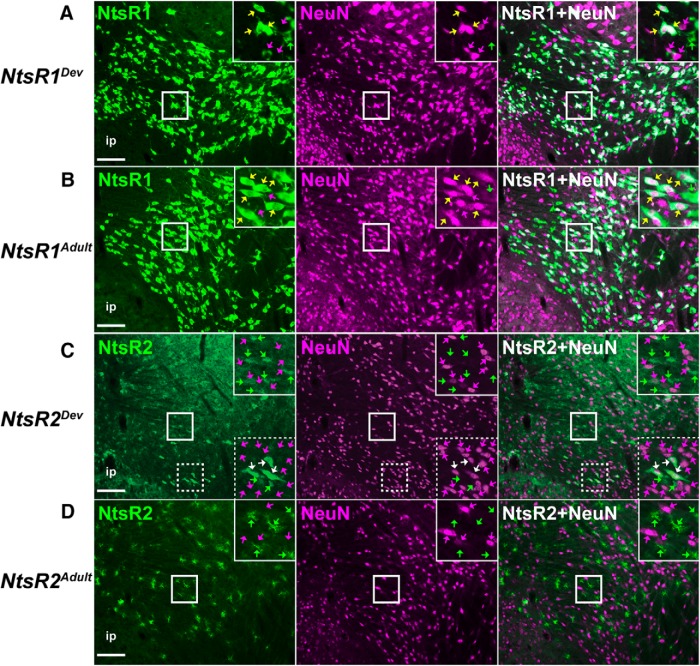
NtsR1 and NtsR2 colocalization with the neuronal marker NeuN in development compared to adulthood. Representative images showing NeuN coexpression in the VTA with (***A***) developmental NtsR1-GFP+ cells, (***B***) adult NtsR1-GFP+ cells, **(*C***) developmental NtsR2-GFP+ cells, and (***D***) adult NtsR2-GFP+ cells. Yellow arrows: NeuN/GFP+ colocalized cells that identify NtsR1 neurons; white arrows: NeuN/GFP+ colocalized cells that identify the modest population of NtsR2 neurons in the VTA; green arrows: GFP+ only cells and magenta arrows = NeuN+ only neurons. Scale bars: 100 µm. Insets are digital enlargements of the indicated boxed areas. *NtsR1^Dev^ n* = 3; *NtsR2^Dev^ n* = 3; *NtsR1^Adult^ n* = 3; *NtsR2^Adult^ n* = 3.

Given the largely dissimilar morphologies of NtsR1-GFP+ and NtsR2-GFP+ cells (Fig. 2), we speculated that these cells might be differentially classed as glia or neurons. To address this we examined VTA sections from the adult and developmental mouse models via immunofluorescence for S100 (a glial marker) and NeuN (a neuronal maker). We observed that GFP+ cells in *NtsR1^Dev^;GFP* mice and *NtsR1^Adult^;GFP* mice were completely distinct from the S100+ glial cells (Fig. 3*A*,*B*). Conversely, most of the GFP+ cells with diffuse, stellate morphology in *NtsR2^Dev^;GFP* mice and *NtsR2^Adult^;GFP* mice overlapped with the S100+ cells (Fig. 3*C*,*D*, yellow arrows), though we also observed spare GFP+ cells with neuronal morphology that did not contain S100 (Fig. 3*C*,*D*). These data suggest that the majority of NtsR2 cells throughout the lifespan are glial in nature. In contrast, essentially all of the GFP+ cells from *NtsR1^Dev^;GFP* mice and *NtsR1^Adult^;GFP* mice colabeled with the neuronal marker NeuN (Fig. 4*A*,*B*, yellow arrows). Since NeuN specifically labels nuclei, the few NtsR1-GFP+ cells apparently lacking NeuN could be due to imaging a plane either above or below the nucleus. We also noted VTA NeuN+ cells that did not contain GFP, indicating that many, but not all, VTA neurons contain NtsR1 (Fig. 4*A*,*B*, magenta arrows). As expected based on our findings from Figure 3, the majority of the glial GFP+ cells in *NtsR2^Dev^;GFP* mice and *NtsR2^Adult^;GFP* mice did not contain NeuN (Fig. 4*C*,*D*). The few GFP+ cells with neuronal morphology in these NtsR2-reporter mice did contain NeuN (Fig. 4*C*, white arrows). In sum, these data confirm that the majority of NtsR2-expressing cells in the VTA are glia, but NtsR1-expressing cells comprise a subset of the total VTA neuronal population.

### NtsR1 is the predominant isoform on VTA DA neurons

**Figure 5.**
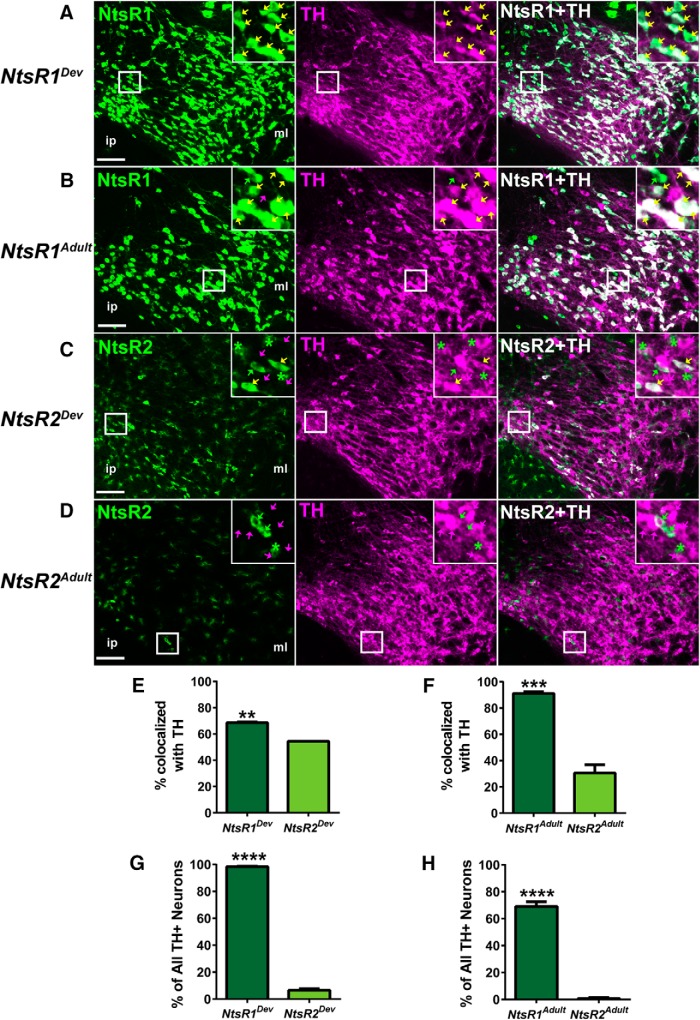
NtsR1 and NtsR2 colocalization with TH in development compared to adulthood. Representative images showing TH coexpression in the VTA with (***A***) developmental NtsR1-GFP+ neurons, (***B***) adult NtsR1-GFP+ neurons, (***C***) developmental NtsR2-GFP+ cells, and (***D***) adult NtsR2-GFP+ cells. Yellow arrows: TH/GFP+ colocalized neurons; green arrows: GFP+ only neurons; magenta arrows: TH+ only neurons; green asterisks: GFP+ glia. Scale bars: 100 µm. Insets are digital enlargements of the indicated boxed areas. Percentage of NtsR1 and NtsR2 neurons that colocalize with TH in (***E***) developmental (*p* = 0.0081_c_) and (***F***) adult expression models (*p* = 0.0003_d_). Percentage of all VTA TH+ neurons that colocalize with NtsR1 or NtsR2 in (***G***) developmental (*p* < 0.0001_e_) and (***H***) adult models (*p* < 0.0001_f_). *NtsR1^Dev^ n* = 3; *NtsR2^Dev^ n* = 3; *NtsR1^Adult^ n* = 3; *NtsR2^Adult^ n* = 6. Bars represent mean ± SEM, and data were analyzed by unpaired *t* tests.

To define the neurochemical phenotype of NtsR1- and NtsR2-expressing neurons, we examined VTA sections from the adult and developmental mouse models via immunofluorescence for tyrosine hydroxylase (TH), the rate-limiting enzyme in catecholamine synthesis and a marker of DA neurons. This analysis revealed that nearly all (∼98%) of VTA DA neurons express NtsR1 at some point in development (Fig. 5*A*,*G*; Table 1), while about two thirds (∼70%) of DA neurons actively coexpress NtsR1 in adulthood (Fig. 5*B*,*H*; Table 1). Interestingly, many TH-negative VTA neurons coexpressed NtsR1 in the developmental model (∼30% of NtsR1-GFP+ neurons) but not the adult model (Fig. 5*E*,*F*; Table 1). Thus, during development NtsR1 is expressed in, and presumably regulates, both DA and other VTA neurons, but only mediates Nts actions in the adult brain via DA neurons. By contrast, the majority of the small population of NtsR2-GFP+ neurons in the developmental and adult models did not colocalize with TH (Fig. 5*C–F*) and NtsR2-GFP expression was found on only 6.5% of DA neurons in *NtsR2^Dev^;GFP* mice and <1% of DA neurons in *NtsR2^Adult^;GFP* mice (Fig. 5*G*,*H*; Table 1). Taken together, these data support the hypothesis that NtsR1 is the dominant isoform regulating VTA DA neurons in both development and adulthood, and suggests that NtsR1 may also modulate non-DA neurons in the VTA during development.

### Projections of VTA NtsR1 neurons

**Figure 6.**
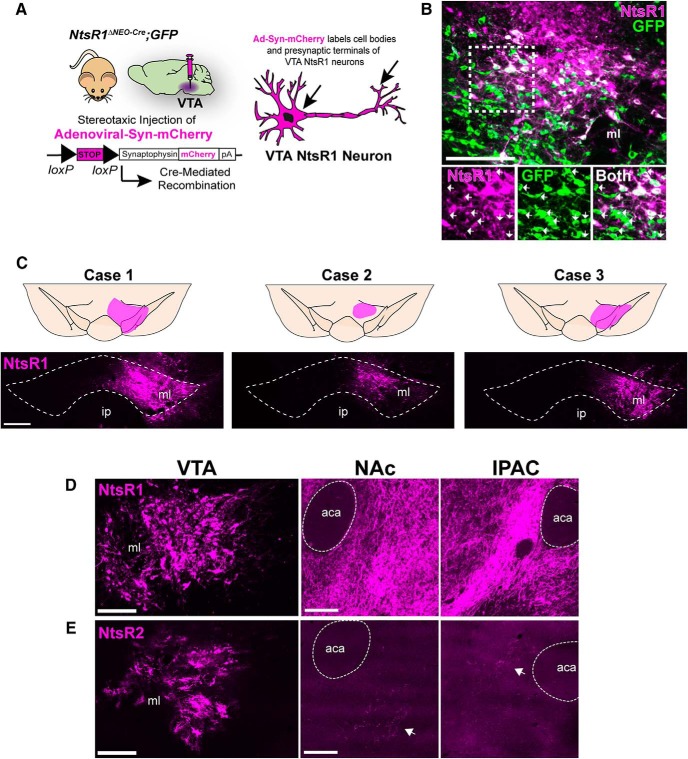
Ad-Syn-mCherry reveals projections of VTA NtsR1 neurons. ***A***, Schematic showing *NtsR1^ΔNEO-Cre^;GFP* mice injected in the VTA with Ad-Syn-mCherry, which labels VTA NtsR1 projections by expression of a Cre-inducible synaptophysin-mCherry fusion protein. ***B***, VTA of *NtsR1^ΔNEO-Cre^;GFP* mouse injected with Ad-Syn-mCherry for 7–10 d showing GFP+ neurons that do not coexpress mCherry (white arrows), representing neurons that transiently expressed NtsR1 during development. ***C***, Distribution of mCherry+ cell bodies within the VTA from 3 individual *NtsR1^ΔNEO-Cre^;GFP* mice 7–10 d after Ad-syn-mCherry injection, confirming selective labeling of VTA NtsR1 neurons. Comparison of VTA injection site and mCherry-labeled terminals in the NAc and IPAC of (***D***) *NtsR1^ΔNEO-Cre^;GFP* mice versus (***E***) *NtsR2^ΔNEO-Cre^;GFP* mice. Scale bars: 100 µm. aca, anterior commissure.

Given that NtsR1 was expressed on some, but not all VTA DA neurons in adult mice, we investigated whether NtsR1 defined the subset of VTA DA neurons that project to the nucleus accumbens (NA) or the prefrontal cortex (PFC; Swanson, 1982), the two major outputs of VTA DA neurons. To do this, we injected adult *NtsR1^ΔNEO-Cre^;GFP* mice in the VTA with the Cre-mediated tract tracer Ad-syn-mCherry and examined brains 7–10 d later (Opland et al., 2013; Fig. 6*A*). In these brains GFP identifies any cell that expressed NtsR1 throughout lifespan, but only cells that actively express NtsR1 can undergo Cre-mediated recombination to express both GFP and the synaptophysin-mCherry fusion protein within cell bodies and terminals (Opland et al., 2013). Thus, this method allows us to discriminate cells that only expressed NtsR1 during development from the adult cells that currently express NtsR1 and can mediate DA release. Visualization of the VTA revealed that nearly all mCherry+ neurons coexpressed GFP; however, many GFP+ neurons within the injection site did not coexpress mCherry (Fig. 6*B*, white arrows). These findings are consistent with our detection of almost twice as many NtsR1-GFP+ neurons in *NtsR1^Dev^;GFP* mice compared to *NtsR1^Adult^;GFP* mice (Fig. 2*E*,*F*) and confirms that NtsR1 expression, and hence induction of Cre, is confined to a limited set of VTA neurons in the adult brain. Out of five injected mice, three had mCherry-labeling confined to the VTA and were used for analysis (Fig. 6*C*). We also injected Ad-syn-mCherry into the VTA of *NtsR2^ΔNEO-Cre^;GFP* mice to verify the minor population NtsR2 neurons in the adult brain and define their projections. In contrast to the numerous mCherry-labeled neurons observed in *NtsR1^ΔNEO-Cre^;GFP* mice (Fig. 4*D*), examination of *NtsR2^ΔNEO-Cre^;GFP* mice revealed very few mCherry+ cells in the VTA. This small population of VTA NtsR2 neurons provided <10 single terminals observed throughout the entire brain, including a minor projection to the NA core (NAc) and the interstitial nucleus of the posterior limb of the anterior commissure (IPAC; Fig. 6*E*, white arrows). These data confirm the dearth of NtsR2-expressing neurons in the adult VTA (Fig. 2*F*,*H*) and support a predominant role for NtsR1 in directly modifying VTA DA signaling.

**Figure 7.**
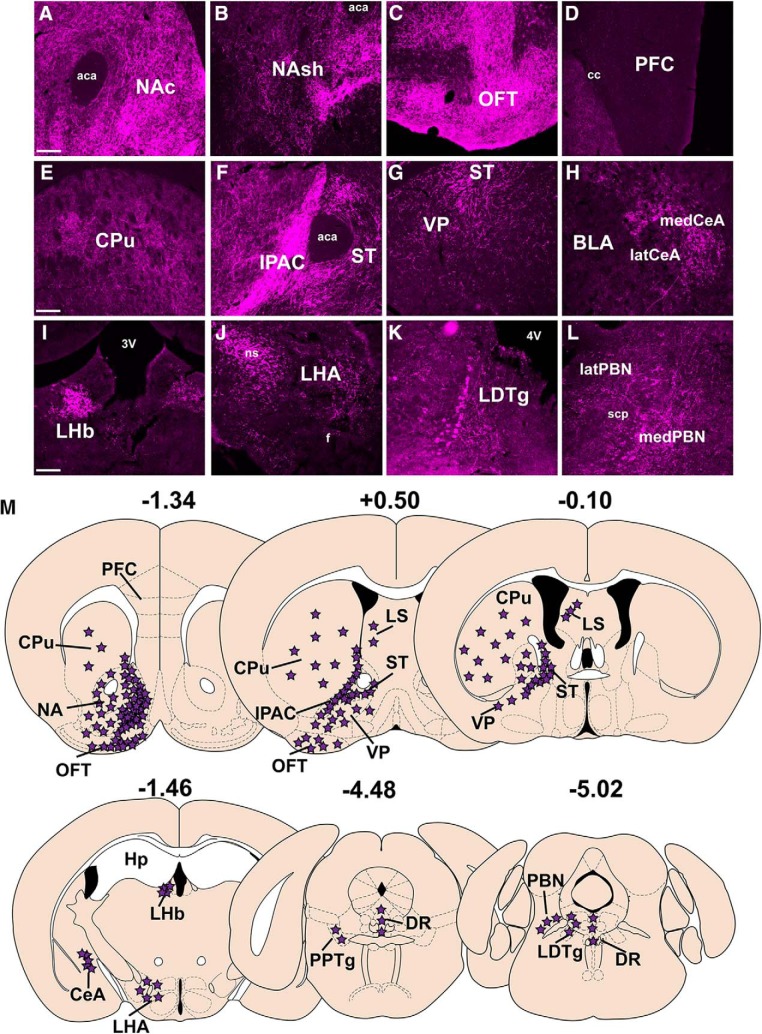
Projections of VTA NtsR1 neurons via acute projection labeling. Distribution of Ad-syn-mCherry-labeled terminals from the 7–10 D-injected mice described in Figure 6. Syn-mCherry-labeled terminals were observed in the (***A***) NAc, (***B***) NAsh, (***C***) OFT, (***D***) absence of terminals in the PFC, (***E***) CPu, (***F***) IPAC and ST, (***G***) VP, (***H***) CeA, both medial (med) and lateral (lat) portions, (***I***) LHb, (***J***) LHA, (***K***) LDTg, and (***L***) the medial and lateral portions of the PBN (medPBN and latPBN). ***M***, Schematic summarizing projections of VTA NtsR1 neurons (purple stars). Scale bars: 100 µm. aca, anterior commissure; cc, corpus callous; BLA, basolateral amygdala; 3V, third ventricle; f, fornix; 4V, fourth ventricle; scp, superior cerebellar peduncle.

We analyzed the projections from the well-targeted *NtsR1^ΔNEO-Cre^;GFP* mice described in Figure 6, and these findings are summarized in Figure 7*M*. We note that our Ad-Syn-mCherry injections were biased to the lateral VTA, but also labeled mCherry+ cell bodies within the medial VTA (Fig. 6*C*), which is known to contain DA neurons that project to the PFC or the NAc (Lammel et al., 2008). Thus, our acute anterograde tracer is appropriately positioned to identify any NtsR1 projections to the NA and PFC, if they exist. We found that VTA NtsR1 neurons most densely project to subregions of the ventral striatum, including the NAc, NA shell (NAsh), and olfactory tubercle (OFT; Fig. 7*A–C*). By contrast, the lack of terminals in the PFC implicates VTA NtsR1 neurons in regulating mesolimbic, but not mesocortical, DA signaling (Swanson, 1982). We also observed dense VTA NtsR1 terminals within the IPAC and modest terminals in the neighboring stria terminalis (ST; Fig. 7*F*). The caudate putamen (CPu) contained sparse VTA NtsR1 terminals (Fig. 7*E*), consistent with the VTA providing less input to the dorsal striatum compared to the SN (Bentivoglio et al., 1979; Swanson, 1982). A few mCherry-labeled terminals were observed in the ventral pallidum (VP; Fig. 7*G*) and the central amygdala (CeA), where DA release is associated with emotional learning and feeding (Guarraci et al., 1999; Rosenkranz and Grace, 2002; Darvas et al., 2011; Anderberg et al., 2014). While we detected few terminals within the lateral hypothalamus (LHA), the dense patch of mCherry+ fibers in the neighboring nigrostriatal tract (ns) likely represents VTA NtsR1 axons traversing the brain en route to the striatum (Fig. 7*J*, see ns). Intriguingly, the lateral habenula (LHb) also contained many mCherry-labeled terminals (Fig. 7*I*), although VTA neurons projecting to the LHb do not release DA and instead inhibit the LHb to indirectly promote reward (Stamatakis et al., 2013). We also observed low to medium terminal density in the hindbrain, namely in the laterodorsal tegmental nucleus (LDTg; Fig. 7*K*), the parabrachial nucleus (PBN; Fig. 7*L*), and the dorsal raphe (DR; Fig. 7*M*).

**Figure 8.**
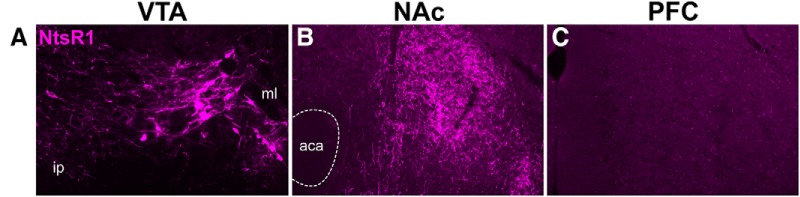
Extended expression of anterograde tract tracer in VTA NtsR1 neurons. *NtsR1^ΔNEO-Cre^;GFP* mice were injected in the VTA with Ad-Syn-mCherry (*n* = 3) and mCherry labeling was assessed four weeks later. ***A***, This extended vector expression time resulted in mCherry labeling of VTA NtsR1 cell bodies and (***B***) numerous projections to the NAc, (***C***) but not to the PFC. aca, anterior commissure.

Our acute anterograde tract tracing suggested that VTA NtsR1 neurons are mesolimbic, but not mesocortical, based on the presence of projections to the ventral striatum and their absence within the PFC. This finding could also result from a technical artifact, for example, if there was insufficient duration of mCherry-tracer expression necessary to label weak and/or far-projecting terminals. While we have previously found that the Ad-Syn-mCherry tracer provides robust projection labeling 10 d post-injection (Opland et al., 2013; Brown, 2017; Woodworth et al., 2017a), we also tested whether longer expression time might identify any VTA NtsR1 projections to the distant PFC. To do this we injected *NtsR1^Cre^* mice (*n* = 3) with Ad-Syn-mCherry and assessed expression four weeks later. This paradigm labeled numerous mCherry+ NtsR1 neurons within the medial and lateral VTA (Fig. 8*A*), which provide significant projections the NAc, but not to the PFC (Fig. 8*B*,*C*). These data suggest that the absence of NtsR1 projections to the PFC is not due to insufficient duration of mCherry-tracer expression necessary to label PFC projections and favor the interpretation that VTA NtsR1 neurons are primarily mesolimbic rather than mesocortical.

## Discussion

Here, we used dual recombinase knock-in mice to identify NtsR1 and NtsR2 cells in the VTA to establish the precise cellular mediators of Nts action. We demonstrate that NtsR2 is predominately expressed on glial cells and only a small number of both DA and non-DAergic neurons throughout life. By contrast, NtsR1 is expressed on many VTA DA neurons during both development and adulthood. Furthermore, adult VTA NtsR1 neurons project to the ventral striatum, not the PFC, and hence are positioned to specifically modify mesolimbic DA signaling. Thus, our data demonstrate NtsR1 as the predominant receptor isoform by which Nts can directly engage DA neurons in the adult brain to modify DA-dependent signaling and physiology.

Previously, NtsR expression was characterized using ISH, which labels mRNA but does not always provide sufficient signal to visualize the morphologic features of the cell in which it is expressed. An advantage of the dual recombinase strategy we used to identify NtsR-expressing cells is that Cre-induced GFP fills the entire cell, which allowed us to clearly distinguish between neuronal and glial morphologies. We were thus able to discern that most NtsR2 is expressed by glial cells throughout the brain, consistent with the diffuse, low intensity signal observed via ISH for *Ntsr2* (Mazella et al., 1996; Sarret et al., 1998; Walker et al., 1998; Asselin et al., 2001). Similarly, cultured astrocytes express NtsR2 (Nouel et al., 1997, 1999; Yamauchi et al., 2007) and Nts modulates their activity (Hösli et al., 1995; Trudeau, 2000). Interestingly, because astrocytes also express endopeptidases that catabolize Nts (Woulfe et al., 1992; Mentlein and Dahms, 1994; Vincent et al., 1994), they might act as a sink to externalize and degrade residual Nts at the synapse via NtsR2. Loss of NtsR2 signaling, as in NtsR2-null mice, could lead to excess synaptic Nts and enhanced Nts-NtsR1 activation of VTA DA neurons, which may explain their increased striatal DA levels and hyperactivity (Liang et al., 2010).

Our findings provide cellular resolution that will be essential to understand how Nts mechanistically acts in the VTA. Consistent with our finding of NtsR1 expression on some VTA DA neurons, the application of Nts or Nts analogues into the VTA activates DA neurons, increases NA DA release, restrains food intake, and promotes reward behaviors, and these effects are reduced by NtsR1 antagonists or developmental deletion of NtsR1 (Remaury et al., 2002; Leonetti et al., 2004; Kim et al., 2008; Kempadoo et al., 2013; Rouibi et al., 2015). Similarly, Bose et al. (2015) suggested that Nts enhances excitatory input onto VTA DA neurons via NtsR1. However, not all studies using pharmacological agents or receptor null mice have supported a predominant role for NtsR1 in directly modulating VTA DA signaling. For example, Kempadoo et al., found that low doses of intra-VTA Nts increased excitatory transmission to DA neurons via NtsR1, while high doses of Nts reduced excitatory input through an NtsR1-independent mechanism, implying involvement of NtsR2 (Kempadoo et al., 2013). Similarly, Rouibi et al., reported that Nts action in the VTA reduces excitatory input to DA neurons via an NtsR1-independent mechanism, again suggesting involvement of NtsR2 (Rouibi et al., 2015). Our finding that <1% of adult VTA DA neurons coexpress NtsR2 suggest it is unlikely that Nts directly modulates most DA neurons via NtsR2. Furthermore, while NtsR2 is expressed on some non DAergic neurons in the adult brain, the overall number is low, which would make them challenging to find using electrophysiology. One possible mechanism to resolve these discrepancies arises from our finding that NtsR2 is predominantly expressed on glial cells, which might act via tripartite synapses to mediate local regulation of DA neurons (Perea et al., 2009). Going forward, the ability to identify NtsR1 and NtsR2 cells will permit direct testing of how Nts engages these cells to orchestrate DA signaling.

While NtsR1 is robustly expressed on VTA DA neurons throughout life, our *NtsR1^NEO-Cre^*; *GFP* models do not distinguish between neurons that express NtsR1 on the soma/dendrites versus synaptic terminals. The subcellular localization of NtsR1 is functionally important because Nts-NtsR1 signaling elicits different behavioral effects via either pre- or postsynaptic mechanisms. Nts-NtsR1 signaling on DA cell bodies in the VTA increases DA release and locomotor activity similar to psychostimulants (Kalivas et al., 1983; Kalivas and Duffy, 1990; Steinberg et al., 1994; Sotty et al., 2000; Legault et al., 2002; St-Gelais et al., 2004). By contrast, Nts-NtsR1 action in the NA suppresses locomotor activity induced by AMPH, cocaine, and DA itself, similar to antipsychotic medications (Ervin et al., 1981; Kalivas et al., 1984; Robledo et al., 1993), and is thought to be mediated by NtsR1 expressed presynaptically on DA terminals in the striatum. Studies using immunohistochemistry and autoradiography suggest that NtsR1 binding sites are present on DA terminals in the ventral and dorsal striatum (Quirion et al., 1985; Dilts and Kalivas, 1989; Schotte and Leysen, 1989). Furthermore, radiolabeled Nts injected in to the striatum accumulates in the cell bodies of VTA DA neurons (Castel et al., 1990, 1992; Faure et al., 1995), suggesting that Nts binds DA neurons at presynaptic terminals and is internalized. Collectively, VTA Nts-NtsR1 signaling appears to have opposing behavioral outcomes depending on whether it occurs pre- or postsynaptically, and this should be taken into account when interpreting future data from *NtsR1^NEO-Cre^* mice.

VTA DA neurons are heterogeneous and have been defined by their projection targets (Juarez and Han, 2016; Morales and Margolis, 2017). We found that adult VTA NtsR1 neurons comprise ∼70% of VTA DA neurons and project primarily to the NA, OFT, and IPAC, but not to other efferent targets of VTA NA neurons such as the PFC or hippocampus (Swanson, 1982). Given that many VTA NtsR1 neurons are mesolimbic, they are likely to modulate the reinforcing properties of natural and pharmacologic rewards, which depend on DA release to the NA (Fiorillo et al., 2003; Rutledge et al., 2010; Lammel et al., 2011, 2012; Hart et al., 2014). Furthermore, our current data provides a cellular and circuit mechanism to explain how Nts signaling directly via VTA NtsR1 neurons can increase NA DA release and mediate conditioned reward (Kalivas et al., 1983; Glimcher et al., 1984, 1987; Sotty et al., 2000; Legault et al., 2002; St-Gelais et al., 2004; Kempadoo et al., 2013; Rouibi et al., 2015). Overall, VTA NtsR1 neurons project to regions that can modify reward intake, suggesting that Nts action via the VTA is necessary for the drive for pharmacological and natural rewards, such as food and social interaction. The functional implications of the VTA NtsR1 projections to regions other than the ventral striatum are less clear, but will be important to investigate in the future.

A limitation of the developmental *NtsR1^ΔNEO-Cre^;GFP* and *NtsR2^ΔNEO-Cre^;GFP* models we developed is that GFP expression does not necessarily reflect cells with active NtsR1 or NtsR2 expression. Inducing Cre expression from the beginning of embryonic development in the developmental models leads to permanent GFP expression in all cells that expressed NtsR1 or NtsR2 at any point in life. Comparing findings from developmental to adult models however, can inform how NtsR expression patterns change through life. For example, essentially all VTA DA neurons colocalize with GFP in *NtsR1^Dev^;GFP* mice whereas only ∼70% colocalize in *NtsR1^Adult^;GFP* mice. These data imply that at some point in development ∼30% of DA neurons transiently expressed NtsR1, but do not identify when they cease expressing NtsR1. Inducing FlpO expression at discrete time points across development will be required to define the temporal dynamics of NtsR1 expression. This could be accomplished by breeding mice to a tamoxifen-inducible FlpO deleter line, whereby injection of tamoxifen would induce Cre expression at any desired point in development (Lao et al., 2012) to label cells actively expressing NtsR1.

Our work indicates vast expression of NtsR1 throughout the brain in *NtsR1^Dev^;GFP* mice, and a heretofore unappreciated potential role for Nts to mediate development of the central nervous system. Within the VTA, *NtsR1^Dev^;GFP* mice showed nearly 100% of VTA DA neurons colocalizing with GFP, which represented ∼65% of all GFP+ neurons. Curiously, this is similar to the overall distribution of DA and non-DA neurons in adults (∼65% TH+, 35% TH-; Morales and Margolis, 2017) and suggests that at some point in life almost every VTA neuron, both DA and non-DAergic, expresses NtsR1. Nts-NtsR1 signaling may thus play a critical role in establishing VTA circuits during development. Mice null for NtsR1 may therefore suffer from abnormal formation of the VTA DA system that causes aberrant DA signaling, locomotor activity, body weight, anxiety, sleep, and exaggerated response to psychostimulants that has been reported to occur in this line (Remaury et al., 2002; Li et al., 2010; Liang et al., 2010; Fitzpatrick et al., 2012; Opland et al., 2013). Our data also identify a substantial population of VTA NtsR1 neurons that persist in the adult brain, and hence signify roles for Nts signaling via NtsR1 in regulating normal physiology. Going forward, using *NtsR1^NEO-Cre^* mice will permit selective manipulation of the VTA NtsR1 neurons in the adult brain to discern how they contribute to DA signaling, physiology, and behavior.

## Synthesis

Reviewing Editor: Christian Lüscher, University of Geneva Decisions are customarily a result of the Reviewing Editor and the peer reviewers coming together and discussing their recommendations until a consensus is reached. When revisions are invited, a fact-based synthesis statement explaining their decision and outlining what is needed to prepare a revision will be listed below. The following reviewer(s) agreed to reveal their identity: Stephan Lammel, Kirill Martemyanov

In this manuscript, the authors generated dual recombinase mice that express FlpO-dependent Cre recombinase in NtsR1 or NtsR2 cells. They found that during development NtsR1 is transiently expressed in nearly all VTA DA neurons and many non-DA neurons. In contrast, in the adult brain only two thirds of VTA DA neurons express NtsR1. Importantly, NtsR2 appears to be largely absent from VTA DA neurons. Lastly, the authors provide data suggesting that NtsR1 is predominantly expressed in mesolimbic, but not mesocortical DA neurons.

Understanding how neurotensin influences VTA DA signaling is critical because previous work has suggested an important role for this neuropeptide in regulating DA release as well as locomotor activity and reward-related behaviors. Altogether, I think this is a very nice set of results further implicating NtsR1 in mesolimbic DA signaling. The mice generated in this study will be important for future investigations into the specific functional role of NtsR1 in VTA DA neurons.

Here are a list of points the authors may want to consider in preparing a revised version.

1. The authors demonstrate lack of synaptophysin mCherry-expressing terminals in the medial PFC, while such mCherry-expressing terminals can be found in the NA. Thus, they claim that “adult VTA NtsR1 neurons project to the ventral striatum, not the PFC, and hence are positioned to specifically modify mesolimbic DA signaling”. Mesocortical DA neurons are predominantly located in the medial VTA (Lammel et al., 2008; Neuron). I noticed that only 1 out of the 3 mice showed mCherry-labeling in the medial VTA (Fig. 4c). Moreover, expressing the construct for 7-10 days is relatively short given that the mesocortical projection is weak and the distance to the medial PFC may be longer compared to the NA. I suggest that the authors increase their sample size and expression time as well as adjust their targeting to more medial parts of the VTA. Moreover, I believe their results will be even stronger if they perform retrograde tracing experiments in order to study NtsR1 expression in the mesolimbic and mesocortical pathways.

2. “Five representative sections spanning the entire VTA were selected from each mouse and were used to determine the number and colocalization of GFP and/or TH positive neurons.” The authors should provide more details on their quantification procedure. How did they select the representative sections? Did they analyze both the anterior and posterior VTA? How did they delineate the VTA? It would also be helpful if they can provide Bregma values. Furthermore, the authors should present sample images for both NtsR1 and NtsR2 expression in the anterior VTA (e.g., at Bregma -2.92 based on the mouse brain atlas by Paxinos and Franklin).

3. “Previously, identification of NtsR1 and NtsR2 neurons has been challenging given the lack of reliable immunohistochemical reagents to visualize them”. I believe the NtsR1 antibody is not as bad as the authors claim it is. In any case, they should support their claim with an appropriate citation.

4. “By contrast, NtsR1Adult;GFP mice had far fewer GFP+ neurons that were primarily restricted to the VTA and SN (Fig. 2B)” There is no SN in Fig. 2B. Although the manuscript focuses on the VTA, the reviewer would appreciate if the authors can also show images of the SN.

5. “We demonstrate that NtsR2 is predominately expressed on glial cells and only a small number of both DA and non-DAergic neurons throughout life”. This claim is based solely on morphological criteria, which might not be reliable. Their claim would be much stronger if they can show either absence of NeuN expression or expression of glial markers (e.g., GFAP) in these cells.

6. Please define ‘IPAC’ in the abbreviations section and in the main text.

7. It may be helpful to examine expression ‘leakage’, if any, in the background strain in the absence of FlpO expression.

8. The need to focus is understandable but the impact and value of the paper could be further increased if expression of NtsRs is surveyed across the entire brain. This may not need to be established with cellular specificity, but knowing which brain regions show enrichment and how VTA compared to them, would be helpful to know.

9. Please consider using markers for glial and neuronal expression in Figure 2, e.g. GFAP, NeuN, Nissl. Simply relying on shape might not be entirely accurate in calling neuron/glia. Perhaps panels E and F could also be combined to make comparisons more straightforward.

10. Figure 3. Given heterogeneity of neuronal populations in the VTA it may be insightful to further break down neurons along excitatory/inhibitory axis and look at co-expression with Gad2 and vGlut1. Also, please consider presenting the data in a pie-chart format so that relative proportion of each population can be estimated.

11. In Figure 4 control injection in Cre-negative brains is missing. This is needed to ascertain specificity of GFP expression in NtsR1+ population.

12. Is it possible to provide some quantification reflecting relative abundance of projections to different areas?

13. Methodologically, how can authors be so sure that injection of adenovirus into lateral ventricles will ensure its equal and efficient spread across the entire brain? Either references to prior studies or controls showing this need to be provided.

14. Given the scope of the report, the Discussion section may be viewed as overly long and unfocused. Please consider trimming it down and limiting it to the most pertinent issues and implications.
